# International children's accelerometry database (ICAD): Design and methods

**DOI:** 10.1186/1471-2458-11-485

**Published:** 2011-06-21

**Authors:** Lauren B Sherar, Pippa Griew, Dale W Esliger, Ashley R Cooper, Ulf Ekelund, Ken Judge, Chris Riddoch

**Affiliations:** 1 College of Kinesiology, University of Saskatchewan, Saskatoon, SK, Canada; 2 School of Sport and Health Sciences, University of Exeter, Heavitree Road, Exeter, UK; 3 Exercise, Nutrition and Health Sciences, School for Policy Studies, University of Bristol, Bristol, UK; 4 Medical Research Council Epidemiology Unit, Cambridge, UK; 5 School of Health and Medical Sciences, Örebro University, Örebro, Sweden; 6 Department for Health, University of Bath, Bath, UK

## Abstract

**Background:**

Over the past decade, accelerometers have increased in popularity as an objective measure of physical activity in free-living individuals. Evidence suggests that objective measures, rather than subjective tools such as questionnaires, are more likely to detect associations between physical activity and health in children. To date, a number of studies of children and adolescents across diverse cultures around the globe have collected accelerometer measures of physical activity accompanied by a broad range of predictor variables and associated health outcomes. The International Children's Accelerometry Database (ICAD) project pooled and reduced raw accelerometer data using standardized methods to create comparable outcome variables across studies. Such data pooling has the potential to improve our knowledge regarding the strength of relationships between physical activity and health. This manuscript describes the contributing studies, outlines the standardized methods used to process the accelerometer data and provides the initial questions which will be addressed using this novel data repository.

**Methods:**

Between September 2008 and May 2010 46,131 raw Actigraph data files and accompanying anthropometric, demographic and health data collected on children (aged 3-18 years) were obtained from 20 studies worldwide and data was reduced using standardized analytical methods.

**Results:**

When using ≥ 8, ≥ 10 and ≥ 12 hrs of wear per day as a criterion, 96%, 93.5% and 86.2% of the males, respectively, and 96.3%, 93.7% and 86% of the females, respectively, had at least one valid day of data.

**Conclusions:**

Pooling raw accelerometer data and accompanying phenotypic data from a number of studies has the potential to: a) increase statistical power due to a large sample size, b) create a more heterogeneous and potentially more representative sample, c) standardize and optimize the analytical methods used in the generation of outcome variables, and d) provide a means to study the causes of inter-study variability in physical activity. Methodological challenges include inflated variability in accelerometry measurements and the wide variation in tools and methods used to collect non-accelerometer data.

## Background

In adults, physical activity is strongly and inversely associated with risk of most major chronic diseases, including obesity, type 2 diabetes, breast and bowel cancer, cardiovascular disease (CVD), musculoskeletal health and psychological well-being [1]. Disease endpoints associated with lack of physical activity are rarely seen in children and it is only in recent years that a consistent association between physical activity and a range of health parameters in children has been described [2]. Understanding the strength of the associations between physical activity and health outcomes has been restricted by difficulties in accurately quantifying physical activity; a complex, multi-dimensional and highly variable behavior. Self-report methods can capture a few sustained activities (e.g. physical education lessons, team sports, etc.) that may be memorable, but children find it difficult to quantify the duration, frequency or intensity of the activity, especially as most children's activity does not occur in sustained bouts - rather, it occurs in numerous short spontaneous bursts of varying intensities [3-5] which are difficult to recall. The quantification of this large volume of 'lifestyle' activity is improved with the use of objective methods. Accelerometers have substantially enhanced our ability to obtain precise measurements of the volume, pattern, frequency, intensity and duration of children's physical activity and sedentary behaviours.

As studies have increasingly adopted objective measurement technologies (e.g., accelerometers) to assess children's physical activity, stronger associations with health outcomes have been identified [6-16]. For example, results from a cross sectional sample (N = 2,049) of British 9-10 yr olds showed a strong inverse graded association between objectively measured physical activity and adiposity and markers of cardiometabolic risk [6]. These findings were consistent across ethnic groups (South Asian, African Caribbean and White European) and mirror results previously found in European children [14]. Results from the Avon Longitudinal Study of Parents and Children (ALSPAC) suggest similar strong relationships between physical activity (also measured by accelerometry) and obesity [13,15] and bone health [16]. Incorporation of accelerometry into large scale surveys has yielded new nationally representative findings. For example, inverse dose-response relations were observed between total physical activity and moderate-to-vigorous physical activity (MVPA) and blood pressure [9] and between medium-to-long bouts of MVPA and BMI [8] in youth (8-17 yrs) from the United States' National Health and Nutrition Examination Survey. Lastly, new insights into the predictors of activity in children have also been identified [17-22]. These findings demonstrate that when large sample size is combined with accurate measurement of outcome and predictor variables, important new insights into the physical activity behaviors of children are obtained.

To date, accelerometer data has been collected in different countries, across diverse cultures, from age ranges encompassing the full childhood and adolescent period, and including a broad range of predictor variables and associated health outcomes. However, because of the multitude of methods used to analyze raw accelerometer files, outcome variables (such as minutes spent in MVPA) are not comparable across studies. Collecting, pooling and reducing raw accelerometer data using standardized methods would create comparable outcome variables across studies. Such data pooling has the potential to improve our knowledge regarding the strength of relationships between physical activity and health outcomes through an increase in statistical power provided by a much larger sample size. Data pooling across studies can also lead to a more heterogeneous (e.g. in terms of ethnicity, culture, socio-economic status, etc.) and thus potentially representative sample. Finally, the study of the causes of variability in physical activity across related contributing studies may also lead to novel insights into socio-cultural and physical environment influences on physical activity.

The aim of this paper is to describe the design and protocols of the International Children's Accelerometry Database (ICAD) project, and how the pooled dataset can be used to address the following main research questions:

1. What are the physical activity levels and patterns of children from diverse geographical backgrounds and how do these vary by age, gender and BMI?

2. What are the dose-response associations between sedentary behaviour, sub-components of physical activity and metabolic health outcomes?

3. To what extent are levels of physical activity in children, and/or the dose response relationship between activity and health outcomes, patterned by measures of socio-economic position in such a way as to provide prima facie evidence of health inequalities?

## Methods

Between September, 2008 and May, 2010, 46,131 raw Actigraph data files and accompanying anthropometric, demographic and health data (where available) collected on children (3-18 yrs, specifically 2.5-18.4 yrs) were obtained from 20 studies worldwide and re-analyzed using standard techniques.

### Recruitment of studies

In the fall of 2008, a pragmatic search for potential contributors was undertaken. Datasets were identified through personal contacts (5 studies) and *Pubmed *searchs (19 studies; using the search-string "accelerometer", "children", "physical activity"). From the search studies which used a version of the Actigraph accelerometer (Actigraph LLC, Pensacola, FL) in children 3-18 years and with sample size > 400 were identified. When a potentially eligible study was identified the Principle Investigator of the study was emailed to gauge general interest regarding pooling of their data. The data sharing policies were communicated at this point.

After a principle investigator or research group had consented for their data to be pooled, contact was made with their data manager to transfer data. Data was transferred using a secured FTP drop-site. Raw accelerometer. DAT files and additional phenotypic information (usually in SPSS, excel or SAS format) were deposited. Contextual information on the methods of data collection and variable coding was also garnered through telephone administered questionnaires.

World-wide, 24 studies were approached and invited to contribute data. The eligibility criteria for inclusion were: 1) physical activity data in the form of raw accelerometer (.dat) files from a version of a waist worn Actigraph accelerometer (e.g. 7164, 71256, GT1M^1^) on children 3-18 years, (2) accompanying data, at a minimum, of gender, age and measured height and weight. Three of the invited groups chose not to contribute data. One further study could not be included because the data did not meet the minimum inclusion criteria. Hence, 20 of the 24 invited studies contributed data. Formal data sharing agreements and policies were established between ICAD and all partners. Each partner consulted with their research ethics board to confirm sufficient ethical approval had been attained for contributing the data. All individual data within the pooled data set were allocated a unique and non-identifiable participant ID to ensure anonymity of data.

### The Actigraph accelerometer

The Actigraph family of accelerometers have become the *de facto standard *for measuring physical activity, having been used in the studies large and small the world over. These Actigraph models are uniaxial^2 ^accelerometers that detect vertical accelerations in the magnitude of 0.05-2.13 g with a frequency response of 0.25-2.50 Hz [23]. These Actigraph accelerometers are small (e.g. GT1M model is 4.5 × 3.5 × 1.0 cm and 43g) and in each of the contributing studies were worn on a belt fastened around the waist. When the Actigraph is accelerated, a voltage signal is generated proportional to the intensity of the acceleration. The acceleration signal is sampled at 10 Hz (7164 and 71256 model) or 30 Hz (GT1M model) and summarized in user-defined intervals (epochs) according to the users specific needs. Once downloaded, these temporally stamped data can be analyzed to provide measures of activity level (volume), and intensity, as well as daily, weekly and seasonal activity patterns [24]. These Actigraph models have demonstrated acceptable levels of technical reliability [25-28]. In addition the Actigraph has been shown to be valid in both children and adolescents [29]. Ekelund et al. [30] assessed the validity of the Actigraph in free-living children using energy expenditure measured by doubly labeled water (DLW) as the criterion measure. Accelerometer output (counts/min) was related to physical activity level (r = 0.58, P < 0.01). This can be compared with correlations of self report versus gold standard measurements that are usually in the range of r = 0.0-0.2 [30].

### Characteristics of the contributing studies

The characteristics of the contributing studies are outlined in Table 1. The majority of the studies are located in Europe (N = 13). The other studies are located in the United States (N = 4), Brazil (N = 1) and Australia (N = 2). Study designs include cross-sectional, longitudinal, closed cohort, and intervention studies. The information provided in Table 1 refers to the data actually deposited in ICAD. For example, in the 1993 Pelotas Birth Cohort Study [31-33] accelerometry was only conducted at one wave (at 13 years) of the longitudinal study and thus, within ICAD, this study is described as cross-sectional. In total, 44,454 viable baseline and repeated measured files were contributed from a total of 31,976 participants (including 12,022 boys and 19,954 girls) aged 2.5-18.4 yrs. With the exception of the female only sample, Project TAAG, the contributing studies collected data on both males and females.

**Table 1:** Details of the 20 studies that contributed data to the International Children's Accelerometry Database (ICAD)

Study Name*		Yrs	Months **	Country	Study design	N Waves	N Subjects	N Files	Age (y)	Model	Epoch	Days***
ALSPAC	[56]	03-07	All	England	Long.	2	6060	10811	10-15	7164, 71256,GT1M	60	7

Ballabeina Study	[57]	08-09	June-Sept	Switzerland	Inter.	2	403	988	4-8	GT1M	15	5

Belgium Pre-School Study	[58-60]	06; 08-09	Oct-March	Belgium	Cross.	1	433	433	3-7	GT1M	15	2-4

CHAMPS (UK)	[61,62]	06-07	Nov-May	England	Cross.	1	562	562	3-17	GT1M	60	7

CHAMPS (US)	[63]	03-06	All	United States	Cross.	1	469	469	3-6	7164	10	4

CLAN^a^	[21,64,65]	01; 04; 06	March-Dec	Australia	Long.	3	1126	2065	5-18	7164, GT1M	60, 20	6

CSCIS	[66,67]	01-05	Oct-May	Denmark	Inter.	2	615	1150	6-11	7164	60	4

EYHS (Denmark)	[68,69]	97-98; 03-04	All	Denmark	Closed Cohort	2	1308	1739	8-18	7164	60	4

EYHS (Estonia)	[69]	98-99	Aug-May	Estonia	Cross.	1	662	662	8-17	7164	60	4

HEAPS	[70,71]	02-03; 06	Feb-Dec	Australia	Long.	2	1362	1714	4-16	7164,GT1M	60	7

Iowa Bone Development Study	[72,73]	98-07	Sept-Dec	United States	Long.	4	437	1996	5-15	7164	60	4-5

KISS	[74]	05-06	May-Nov	Switzerland	Inter.	2	433	909	6-14	7164, GT1M	60	7

MAGIC	[75]	02	Sept-Oct	Scotland	Cross.	1	462	462	4-5	7164	60	6

NHANES	[8,9,76,77]	03-04; 05-06	All	United States	Cross.	2	5174	4970	6-18	GT1M	60	7

EYHS (Norway)	[69,78]	99-00	Feb-June; Oct	Norway	Cross.	1	391	391	9-10	7164	60	4

PEACH	[79]	06-09	Sept-July	England	Long.	2	1232	2091	10-13	GT1M	15	7

Pelotas 1993 Birth Cohort	[31-33]	06-07	Aug-Feb	Brazil	Cross.	1	457	457	13-14	GT1M	5	4

Portugal EYHS	[69,80]	99-00	Jan-July	Portugal	Long.	2	1242	1386	8-18	GT1M	60	4

Project TAAG	[81,82]	02-06	Oct-May	United States	Closed Cohort Inter.	2	7148	8995	10-17	7164	30	7

SPEEDY	[7,6,83]	07	Feb-July	England	Cross.	1	2000	2000	9-11	GT1M	5	7

						Total	**31976**	**44544**				

Note: *See Additional file 1, Table S8 for full name of study; ** Approximate months during which data collection (across all waves) occurred; *** Days = Number of days of Actigraph deployment. N Files = the total number of files contributed from each study that are present in the database (excluding spurious and 'temporally shifted' files); Long - Longitudinal; Cross = Cross-sectional; Inter. = Intervention. Note: the number of participants will differ from the number of files when a study has repeated measures on the same individual (e.g. longitudinal and or closed cohort design); ^a ^Baseline wave of CLAN was named CLASS ("Children's Leisure Activities Study") in early publications.

Data were analyzed using KineSoft version 3.3.20 (KineSoft, Saskatchewan, Canada; http://www.kinesoft.org).

### Accelerometer Data Cleaning

**Figure 1:**
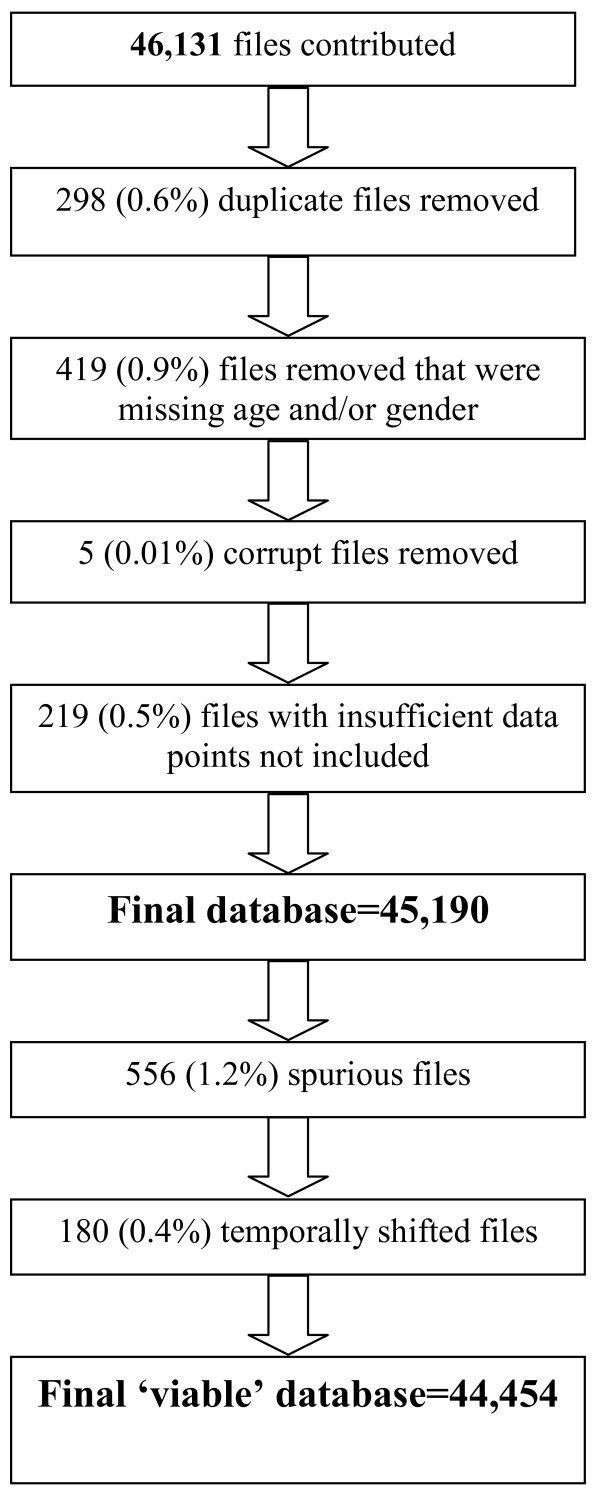
**The final sample size of the pooled database**.

Partners were requested to contribute all raw Actigraph data files (i.e. files with a. dat file extension). All studies were able to meet this request with the exception of the NHANES studies (see below for discussion of the special treatment for this study). In total 46,131 files were deposited (Figure 1). Of these, 298 (0.6%) duplicate files for the same participant were removed. This occurred when two monitors were placed on an individual, either as a part of a validation study or to increase the chances of obtaining a reliable file (i.e. if one monitor fails, there will be data from another monitor). In these instances the files were viewed and if one was corrupt, the non-corrupt file was used. If both files were 'not corrupt' then the first labeled file was used. A total of 419 (0.9%) files were not included because they did not have the minimum accompanying variables. Finally, 219 (0.5%) files deemed to have no wear time data (discussed in detail below) and 5 (0.01%) corrupt files were unable to be processed and were not included in the database. This resulted in the processing of 45,190 accelerometer files.

**Figure 2:**
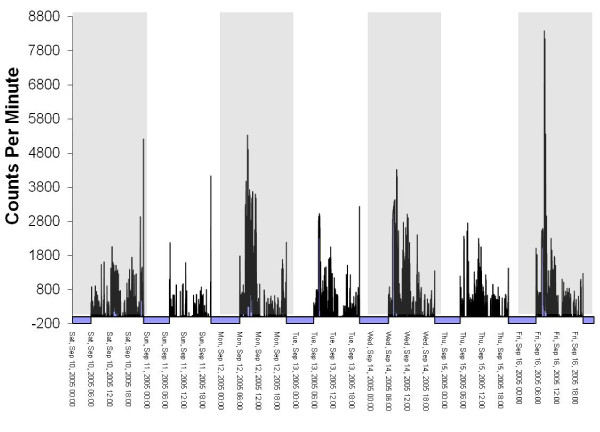
**Graphical example of an 'OK' file**.

**Figure 3:**
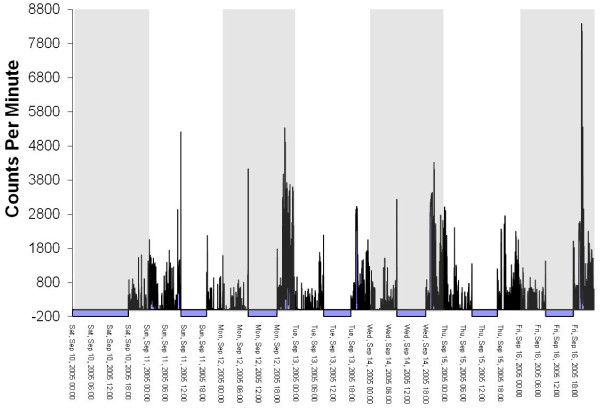
**Graphical example of a 'temporally shifted' file**.

**Figure 4:**
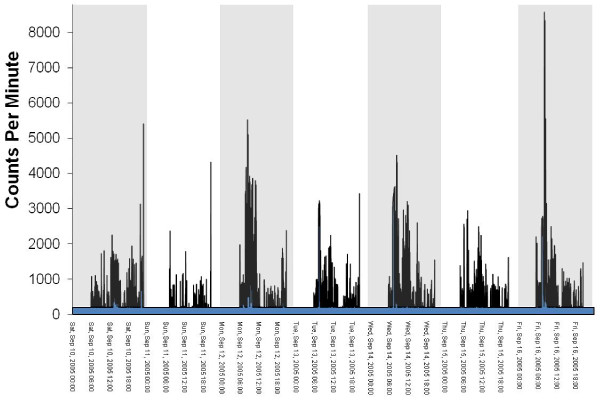
**Graphical example of a potentially spurious file that does not return to baseline (zero)**.

**Figure 5:**
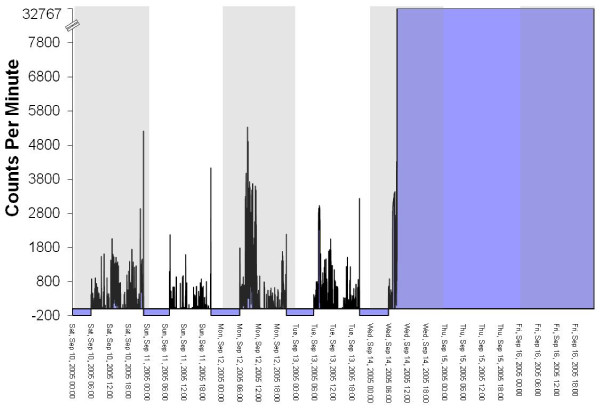
**Graphical example of a file from a malfunctioned unit where the sensor voltage plateaus at saturation (32767 counts)**.

The files in the database were 'flagged' if they had one of more of the following characteristics (see Figure 2 for an example of an 'OK' file and Figures 3, 4 and 5 for examples of files flagged as spurious):

1. Overnight wear of ≥ 10 minutes at hrs 2, 3 and/or 4 am. Visual graphing of these files was used to determine the reason for the overnight wear. The four reasons for overnight wear were: i) Legitimate wearing of the monitor overnight, ii) 'Temporally shifted' file (Figure 3), iii) Potentially spurious device that does not return to baseline (zero) (Figure 4), iv) Malfunctioned unit (Figure 5). A 'temporally shifted' file is when the time stamping of the data is shifted by a number of hours which results in greater than expected consecutive zeros during the day and activity counts during the night on each day of monitoring.

2. Plateau (3 consecutive counts at the same number) at a count ≥ 10. This was a good indicator of technical faults with the device (with plateaus occurring most often at 32767 counts) (Figure 5).

A variable was included in the database which indicates files considered spurious (556 (1.2%)) and 'temporally shifted' (180 (0.4%)). It is recommended that these files are excluded from further analysis. Thus the final database includes data obtained from 44,454 'viable' accelerometer files (Figure 1).

### Non-accelerometer data

To accompany the accelerometer files, all projects contributed additional participant information. Studies were included in ICAD if they had a minimum of age, gender, height and weight. However, within a study, individuals were included if they had age and gender at a minimum (i.e. could be missing height and weight). Where available, additional information including body composition (e.g. circumference measures, skinfolds), health markers (e.g. blood pressure, cholesterol), economic indicators (e.g. household income), parental information (e.g. height, weight) and behavioral variables (e.g. school travel mode, TV viewing) were also requested.

For a variable to be included in the database, it had to be present in at least 3 contributing studies. The coding procedure for all variables was standardized across projects to ensure uniformity within the database. Where necessary, the appropriate formulae were used to re-calculate the unit of measurement for body composition variables. To create standardized categorical variables the response categories used by different projects were compared and, where appropriate, combined category options were created for ICAD. Original participant responses were then re-coded to the new ICAD categories. See Additional file 1 for a full description of all non-accelerometer variables and further re-coding details.

### Final sample characteristics

**Figure 6:**
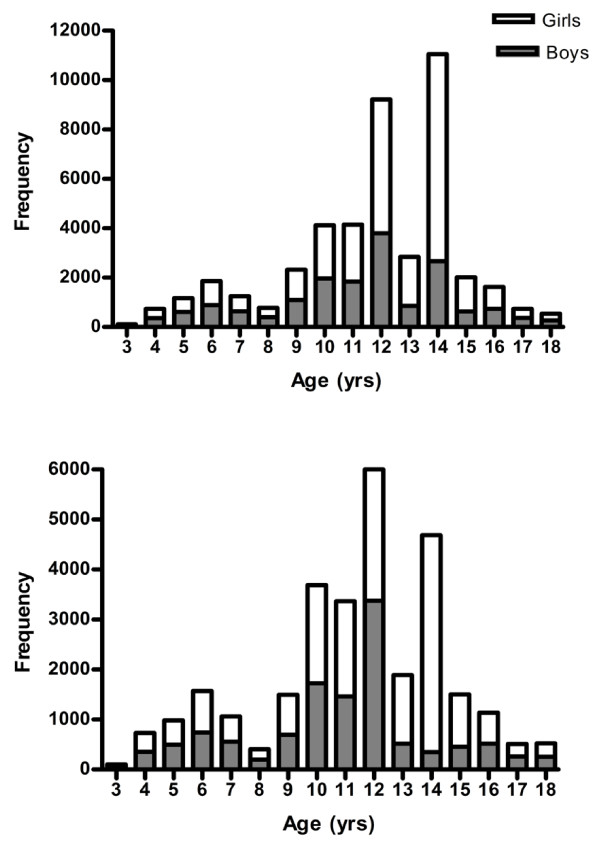
**Sample size distribution; a) with repeated measures included; b) baseline only data**.

Figure 6 shows the sample size at each chronological age for girls and boys. The numbers include all the viable data (i.e. excluding spurious and temporally shifted files) in the final database (total = 44,454). Figure 6a shows the age distribution of all files, including repeated measures on the same individual. Figure 6b shows the distribution of the baseline measures (N = 31,976) only (thus participants are only featured once). At most ages there is a fairly equal representation of girls and boys with the exception of age 13 and 14, where more females are represented. This is primarily due to the contribution of data at these ages from Project TAAG, a female-only study.

### Data Collection Information

The 20 contributing studies collected accelerometer data using three versions of the Actigraph family of monitors (see Table 1). Of the 20 studies, the Actigraph (7164, also known as the CSA and MTI) was used in 8, the newer Actigraph GT1M, model was used in 8, and 4 studies used a mixture of both models including one where the 71256 model was used (which only differs from the 7164 with respect to the size of the memory).

The fact that epoch length influences the interpretation of accelerometer data has been well documented [34-40]. An epoch, refers to the amount of time over which activity counts are summed and stored. Epochs varied from 5 seconds to 60 seconds (see Table 1). The older studies tended to collect at 60 second epochs, because the older generation accelerometers were only capable of storing data collected using epoch lengths of < 60 seconds for a limited number of days. Newer generations of the Actigraph have increased storage capacity allowing for higher resolution data to be collected while still being able to measure physical activity for at least 7 days. For the purpose of the pooled dataset it was necessary that all outcome variables be comparable; therefore, all files which used an epoch < 60 seconds were reintegrated up to 60 seconds for analysis using an automated tool in the KineSoft toolbox.

### Data reduction

#### Determination of accelerometer wear and non-wear

The decision was taken to consider accelerometers as not worn if a period of 60 minutes of consecutive zeros, allowing for 2 minutes of non-zero interruptions, was encountered anywhere in the data array. All other count data contributed to the determination of accelerometer wear time. Non-wear data received special coding in order to separate and exclude the non-wear zeros from the legitimate zeros that often occur during periods of sedentary behavior. No imputation or data modeling was used to replace missing data.

#### Nhanes

Although data from the NHANES study are publically available, the raw Actigraph data are not. The data are only available in the Statistical Analysis Software (SAS) format and important aspects of the Actigraph file header, such as the serial number, initialization date, initialization time, download date, and download time, are not made available due to confidentiality concerns. Therefore, the SAS formatted, time-stamped count data was reconstituted into individual raw Actigraph. dat files with start times and epoch lengths of 00:00:00 and 60 seconds, respectively. Fortunately, NHANES used semi-automated initialization procedures so human errors were not possible. The file start dates were adjusted from January 1st thru 7th in order to maintain the correct day of the week of the reconstituted. dat files. The year of the start date was listed as 2004 for all files in the 2003-04 NHANES dataset and was listed as 2006 for all files in the 2005-06 NHANES dataset.

#### Daylight Saving or Summer Time Event

When Actigraph files spanned a daylight saving time (DST) or summer time event a simple data modeling tool was used to ensure all files would be compared equally. In the spring, when the clocks were set ahead, the missing hour of data was imputed based on the replication of the 30 minutes pre and post the event time. In the fall, when clocks were set back, the middle hour epoch was used only.

#### Start dates

The vast majority of Actigraph data files were initialized to collect data on day one of the instrument's deployment; however, in a minority of cases, Actigraphs were initialized prior to being deployed. In these cases, the start date and/or start time was altered using a read-in-file with the file specific date and/or time exceptions. This analytical functionality was important as it allowed the studies to be processed in large batches.

#### Physical activity outcome variables

One frequently used accelerometer post-processing method involves the use of physical activity intensity thresholds (i.e., cutpoints based on count values) to summarize time spent in a given intensity category (e.g., sedentary, light, moderate and vigorous). There are many cutpoints that have been published for use with the Actigraph accelerometer [29,41-47]. At present there is no firm consensus regarding the optimal intensity cutpoints to use when analyzing the Actigraph data of children, adolescents or adults. However, a number of cutpoints have achieved a level of acceptability. In an effort to provide researchers with physical activity data derived from a range of Actigraph cutpoints, variables were derived using multiple cutpoints (see Additional file 2, Table S5). It is expected that as the database evolves, new cutpoints will be added as the field progresses.

How time spent in different intensities is accumulated will also be provided in ICAD. For example, MVPA accumulated in bouts lasting 0-10 and 10+ minutes will be provided in the database. For full listings of the bouts provided for each intensity see Additional file 2, Table S4. Furthermore, data (for all intensity variables) will be provided for specific windows of time. The analytical software was prompted to provide 1 hr (on the hour) windows (hr1-hr24). In addition other windows of interest (determined by scrutinizing the literature and obtaining feedback from partners) were also provided, including before or after school periods, morning and afternoon commute, and lunch and recess time (see Additional file 2, Table S3). A data dictionary which provides a definition of all accelerometer variables in the ICAD database can be found at http://www.mrc-epid.cam.ac.uk/Research/Studies/

## Results

Among the males 96%, 93.5% and 86.2% had at least one valid day of data when using ≥ 8, ≥ 10 and ≥ 12 hrs of wear as a criteria for a valid day (Table 2). Among the females 96.3%, 93.7% and 86% had at least one valid day of data when using ≥ 8, ≥ 10 and ≥ 12 hrs of wear as a criteria for a valid day (Table 3). The number of valid days of data are less in the pre-school ages (3-5 years) as compared to the older ages (6-18 yrs), which can be partly explained by a shorter deployment time (2-5 days) among the pre-school studies.

**Table 2:** Percentage of male respondents, by valid days of accelerometer wear (using ≥ 8, ≥ 10 and ≥ 12 hours of wear as criteria) and age group

	Number of valid days									
	**0**	**1**	**2**	**3**	**4**	**5**	**6**	**7**	**≥ 1**	**≥ 4**

**3 to 5**										

≥ 8 hrs	5.7	8.2	19.8	25.5	23.1	8.5	8.1	1.1	**94.3**	**40.8**

≥ 10 hrs	10.4	12.7	23.1	25.9	18.5	5.4	3.8	0.2	**89.6**	**28.0**

≥ 12 hrs	27.8	23.6	22.2	18.1	6.9	1.2	0.1	0.2	**72.2**	**8.3**

**6 to 8 **										

≥ 8 hrs	2.9	3.4	5.3	9.7	33.0	11.8	16.3	17.7	**97.1**	**78.7**

≥ 10 hrs	4.9	5.7	8.1	15.6	29.3	10.2	13.1	13.1	**95.1**	**65.7**

≥ 12 hrs	11.1	13.3	18.7	17.9	16.6	8.8	5.7	7.9	**88.9**	**39.0**

**9 to 11 **										

≥ 8 hrs	1.5	2.5	4.4	9.0	29.2	20.0	20.9	12.5	**98.5**	**82.6**

≥ 10 hrs	2.7	3.9	6.4	12.9	29.6	18.9	16.9	8.6	**97.3**	**74.0**

≥ 12 hrs	6.3	8.1	14.8	21.7	23.1	13.3	8.2	4.4	**93.7**	**49.1**

**12 to14 **										

≥ 8 hrs	3.7	2.9	3.6	6.2	11.5	19.2	18.9	34.0	**96.3**	**83.6**

≥ 10 hrs	5.5	3.7	5.6	8.9	14.2	18.8	19.0	24.4	**94.6**	**76.4**

≥ 12 hrs	8.9	7.2	9.2	12.5	19.0	17.5	15.8	9.9	**91.1**	**62.2**

**15 to18 **										

≥ 8 hrs	6.0	5.6	6.7	10.9	19.1	17.0	18.5	16.2	**94.0**	**70.7**

≥ 10 hrs	9.2	7.4	9.4	12.6	18.9	17.4	14.4	10.7	**90.8**	**61.4**

≥ 12 hrs	14.8	11.0	12.4	16.2	16.6	13.3	9.8	5.8	**85.2**	**45.5**

**Overall **										

8 hrs	**3.9**	**4.5**	**8.0**	**12.3**	**23.2**	**15.3**	**16.5**	**16.3**	**96.0**	**71.3**

10 hrs	**6.6**	**6.7**	**10.5**	**15.2**	**22.1**	**14.2**	**13.4**	**11.4**	**93.5**	**61.1**

12 hrs	**13.8**	**12.6**	**15.5**	**17.3**	**16.5**	**10.8**	**7.9**	**5.6**	**86.2**	**40.8**

**Table 3:** Percentage of female respondents, by valid days of accelerometer wear (using ≥ 8, ≥ 10 and ≥ 12 hours of wear as criteria) and age group

	Number of valid days									
	**0**	**1**	**2**	**3**	**4**	**5**	**6**	**7**	**≥ 1**	**≥ 4**

**3 to 5**										

≥ 8 hrs	7.7	8.6	20.8	19.9	25.0	9.0	8.4	0.7	**92.3**	**43.1**

≥ 10 hrs	12.8	11.9	24.3	21.2	21.1	5.0	3.4	0.3	**87.2**	**29.8**

≥ 12 hrs	30.6	23.2	24.0	14.0	6.6	1.0	0.4	0.1	**69.3**	**8.1**

**6 to 8 **										

≥ 8 hrs	2.3	3.1	5.8	9.7	31.3	13.7	15.5	18.6	**97.7**	**79.2**

≥ 10 hrs	4.4	5.0	10.3	14.2	29.5	10.6	11.7	14.2	**95.6**	**66.1**

≥ 12 hrs	11.6	12.8	17.7	19.5	15.8	7.2	6.0	9.4	**88.5**	**38.4**

**9 to 11 **										

≥ 8 hrs	1.0	1.9	3.8	8.2	29.0	19.7	22.8	13.6	**99.0**	**85.1**

≥ 10 hrs	1.9	3.2	6.5	12.8	29.5	19.7	17.9	8.6	**98.1**	**75.6**

≥ 12 hrs	5.2	8.3	14.8	20.6	24.8	14.5	8.0	3.9	**94.8**	**51.1**

**12 to14 **										

≥ 8 hrs	2.6	3.2	4.3	7.8	11.8	18.3	24.8	27.1	**97.4**	**82.0**

≥ 10 hrs	4.3	4.7	6.6	9.9	15.7	19.3	22.6	16.8	**95.7**	**74.4**

≥ 12 hrs	7.6	8.3	10.3	14.5	19.2	18.8	15.2	6.2	**92.4**	**59.3**

**15 to18 **										

≥ 8 hrs	5.2	6.4	6.3	9.6	19.2	19.4	19.0	15.1	**94.9**	**72.6**

≥ 10 hrs	8.0	9.0	9.0	12.9	20.5	15.3	16.3	9.1	**92.0**	**61.1**

≥ 12 hrs	14.8	11.2	13.0	16.3	18.0	13.4	9.3	4.1	**85.2**	**44.7**

**Overall **										

8 hrs	**3.8**	**4.6**	**8.2**	**11.0**	**23.3**	**16.0**	**18.1**	**15.0**	**96.3**	**72.4**

10 hrs	**6.3**	**6.8**	**11.3**	**14.2**	**23.3**	**14.0**	**14.4**	**9.8**	**93.7**	**61.4**

12 hrs	**14.0**	**12.8**	**16.0**	**17.0**	**16.9**	**11.0**	**7.8**	**4.7**	**86.0**	**40.3**

## Discussion

### Selection of studies

In terms of meta-analyses it is necessary to include all relevant studies, or at least a representative sample, of which only clearly flawed studies or studies with small sample size are omitted. These same sampling considerations could be applied to this study. However, although a large number of studies have contributed to ICAD, some smaller studies and studies that had not published any findings on *PubMed *may have been missed.

### Variability between studies

As previously mentioned, an obvious advantage of pooling accelerometer data is a very practical one: to increase sample size. However, this comes at a price: in comparison to a single large-scale study, ICAD will have inflated variability in its accelerometer measurements. This is because, although much variability is reduced by the utilization of standardized analytical techniques, variation in the deployment strategies used between contributing studies can generate significant between-study variability in accelerometer outputs. One potential cause of between-study variability is the use of different models (i.e. generations) of the Actigraph. In fact Rothney et al. 2008 [48] found differences in the outputs from the 7164 and the GT1M monitors when undergoing mechanical testing. Unfortunately, understanding these differences is hampered by the fact that the unlike the Actigraph 7164 (see [23]) there is no published paper detailing the specifications of the Actigraph GT1M. Fortunately, recent studies [28,49-51] provide insight into technical specifications of the GT1M. Despite the between model differences in the Actigraph, studies in free-living individuals showed that during self-paced locomotion at a range of speeds, the different Actigraph models did not result in meaningful differences in the classification of physical activity intensity [50] or activity count output [49]. Increased variability could also be caused by variation in the season (e.g. spring, summer, winter, fall) in which the accelerometer was worn [52,53].

The above limitations notwithstanding, the increased variability in ICAD can be considered a real advantage. For example, the main strength of the pooled ICAD database is its enhanced social and cultural diversity, compared to any of the individual studies. The investigation of the influence of these socio-cultural influences may lead to new insights into how children's physical activity patterns are shaped. Furthermore, including participants from a variety of geographical locations may lead to further insights into the possible influence of the physical environment. Lastly, analysis of pooled data can be useful to quantitatively examine the generalizability of findings. For example, findings that are replicated across individual studies carry increased validity, as they are more likely to be linked to the research question being addressed than to differences in study design and data collection procedures (such as different climatic zones, different seasons, different days of the week, different number of days of deployment, etc.).

### Non-accelerometer data

Unfortunately, with the large variation in the tools used to assess nutrition and psychological constructs, these data are not suitable for pooling and were not collected. This is a limitation of the database. If repositories of accelerometer data become more mainstream investigators may see added benefit of considering measures that are consistent between large studies (as seen in recent projects such as the Public Population Project in Genomics (P3G) [54]). Furthermore, this may accelerate the work aimed at equalizing scales and measures, thus enabling analysis of nutrition and psychological data, as it relates to physical activity, in the future.

### Data Availability

The ICAD partners (i.e. contributors of the data) have sole access to the pooled database for 12 months (March 2011-Feb 2012), after which time the database is open to any approved user (see http://www.mrc-epid.cam.ac.uk/Research/Studies/ for more information on the procedures for using ICAD).

## Conclusions

Increasing physical activity levels in those insufficiently active is a complex and long term issue. We currently do not have sufficient evidence to design effective physical activity interventions that will result in long-term behaviour change. In essence, we do not know the full range of determinants of physical activity, nor do we have sufficient knowledge of how different levels and patterns of activity are associated with physiological and psychological health outcomes. Public health requires a more substantial and comprehensive understanding of this complex behavior [55]. Pooling objective measurements of physical activity and accompanying physiological, demographic and health data from diverse studies in contrasting settings maximizes analytical power and provides new evidence on potential social, cultural and environmental influences on behaviour. Such a database can potentially give important insights into physical activity and health inequalities. It is hoped that ICAD will be a valuable resource for researchers, policy makers and practitioners, in their search for evidence, as well as less senior researchers and students who might use the resource as part of their training program. We hope that the ICAD database will be further developed so that new accelerometer studies can be added (for example studies of adults) and become a publically available archive.

## Competing interests

Dale Esliger is the owner and founder of KineSoft, the accelerometer data analysis software used for the creation of the accelerometer outcome variables deposited in the ICAD.

## Authors' contributions

LBS recruited studies, cleaned and reduced the raw accelerometer data to the outcome variables, wrote the first draft of the manuscript and developed Additional file 2. PG organized the transfer of data, compiled and re-coded the non-accelerometer variables, merged data to form the final database and developed Additional file 1. DWE consulted on accelerometer data reduction and helped draft the manuscript. AC and UE contributed to the conceiving the project, participated in the design and coordination of the project and in the reduction of the data. KJ participated in the design and coordination of the project and consulted in re-coding the socio-demographic data. CR contributed to the conceiving of the project, coordinated the project, participated in its design and helped to draft the manuscript. All authors read and approved the final manuscript.

## Footnotes

^1^Note: The most recent Actigraph model (the GT3X) had not been released at the time data were deposited

^2^None of the GT1M accelerometers used in ICAD underwent the firmware update to activate the second (i.e. dual) axis which was introduced as an option in 2008

## Pre-publication history

The pre-publication history for this paper can be accessed here:

http://www.biomedcentral.com/1471-2458/11/485/prepub

## Supplementary Material

Additional file 1 **Non-Accelerometer Variables included in the International Children's Accelerometer Database (ICAD)**. Click here for file

Additional file 2 **Accelerometer Variables included in the International Children's Accelerometer Database (ICAD) **[84-86] . Click here for file
