# Supplementary material for: International children's accelerometry database (ICAD): Design and methods
Source: BMC Public Health. 2011 Jun 21;11:485. doi: 10.1186/1471-2458-11-485 (PMC3146860; doi:10.1186/1471-2458-11-485)
Supplement: Additional file 1 — Non-Accelerometer Variables included in the International Children's Accelerometer Database (ICAD). [file 1471-2458-11-485-S1.PDF]

Additional File 1

Non-Accelerometer Variables included in the International Children's Accelerometer Database (ICAD)

The tables below describe the non-accelerometer variables in the ICAD database. Included are a description of the coding of the variables and the studies that provide each variable.

Table S1: Identifiers and project details

ICAD Variable	Variable Descriptor	Coding	Studies*
icad_id	Participant ID	A new ICAD specific non-identifiable participant ID was assigned	ALL
icad_wave	Wave of data collection	1 – wave 1 (baseline)<br>2 – wave 2<br>3 – wave 3<br>4 – wave 4	ALL
Study	Study ID	A new ICAD specific study ID was assigned	ALL
icad_project_id	Study and wave ID	A new ICAD specific Study and wave ID was assigned	ALL
icad_design	Study design	1- Cross-sectional<br>2- 2- Longitudinal<br>3- 3- Intervention	ALL
icad_int	Participant assignment within an intervention	1- Intervention<br>2- Control	4, 17

icad_country	Country where study was conducted	1-Austrailia<br>2-Belgium<br>3-Brazil<br>4-Denmark<br>5-Estonia<br>6-Iceland<br>7-Portugal<br>8-United Kingdom<br>9-United States<br>10-Switzerland	ALL

* Studies that provided the variable. See key (Table S8) for each study code (1-21)

Table S2: Demographics

ICAD Variable Name	Variable Descriptor	Coding/Unit	Studies*
icad_age	Age of study participant at measure (yrs)	Calculated (yrs, one decimal place)	ALL
icad_gender	Participant gender	0-boy<br>1-girl	ALL
icad_dob	Date of birth	dd.mm.yy	2, 3, 4, 7, 9, 13, 14, 16, 17, 18, 19, 20
icad_dom	Date of measure	dd.mm.yy	2, 3 , 4, 7, 9, 13, 14, 16, 17, 19
icad_ethnicity	Ethnicity	1-White<br>2-Black<br>3-Asian<br>4-Hispanic<br>5-Mixed<br>6-Other	5, 6, 8, 10, 11, 12, 13, 15, 17, 18
icad_white	Ethnicity	1- White<br>2- Other	1, 5, 6, 8, 10, 12, 13, 14, 15, 16, 17, 18

* Studies that provided the variable. See key (Table S8) for each study code (1-21)

Table S3: Body Composition

ICAD Variable Name	Variable Descriptor	Coding	Studies*
icad_height	Participant height (measured)	CM	ALL
icad_weight	Participant weight (measured)	KG	ALL
icad_waist_C	Waist circumference	CM	1, 4, 5, 6, 9, 10, 11, 12, 13, 14, 15, 16, 19, 20
icad_thigh_c	Thigh circumference	CM	10, 12, 14
icad_hip_c	Hip circumference	CM	1, 4, 5, 6, 11, 14, 15
icad_arm_c	Arm circumference	CM	1, 9, 10, 12, 14
icad_triceps_s	Skinfold - triceps	MM	4, 5, 6, 10, 11, 12, 14, 15, 17
icad_biceps_s	Skinfold - biceps	MM	4, 5, 6, 11, 15
icad_subscapular_s	Skinfold - subscapular	MM	4, 5, 6, 10, 11, 12, 14, 15
icad_suprailiaca_s	Skinfold - suprailiac	MM	4, 5, 6, 11, 15
icad_ps_pubic	Pubic hair development	Stage 1 – 5	1, 4, 5, 6, 8, 11, 15, 20
icad_ps_brest	Breast development	Stage 1 – 5	1, 4, 5, 6, 8, 11, 15, 20
icad_ps_genital	Genital development	Stage 1 – 5	1, 4, 5, 6, 8, 11, 15
icad_ps_mencarche	Recalled age at menarche	Years	1, 8, 10, 12, 13, 14

* Studies that provided the variable. See key (Table S8) for each study code (1-21)

Table S4: Health Markers

ICAD Variable Name	Variable Descriptor	Coding	Studies*
icad_fasting_blood	Were blood samples taking when fasting?	1 – yes<br>2 – no	4, 5, 6, 10,11, 12, 15, 20
icad_bp_diastolic	Diastolic blood pressure	Mm Hg	1, 4, 5, 6, 9, 10, 11, 12, 14, 15
icad_bp_systolic	Systolic blood pressure	Mm Hg	1, 4, 5, 6, 9, 10, 11, 12, 14, 15
icad_chol_hdl	HDL cholesterol	Mmol,l	4, 5, 6, 10, 11, 12, 15, 20
icad_chol_ldl	LDL cholesterol	Mmol,l	4, 5, 6, 10, 11, 12, 15, 20
icad_triglycerides	Triglycerides	Mmol,l	4, 5, 6, 10, 11, 12, 15, 20
icad_glucose	Glucose	Mmol,l	4, 5, 6, 10, 12, 15, 20
Icad_insulin	Insulin	pmol/l	4, 5, 6, 10, 12, 15, 20
icad_birth_weight	Participant birth weight	Grams	1, 5, 6, 8, 11, 14, 15, 16, 20

* Studies that provided the variable. See key (Table S8) for each study code (1-21)

Table S5: Economic indicators

ICAD Variable	Variable Descriptor	Coding	Studies*
icad_house_inc	Household income quartiles	1- Lowest<br>4- Highest	1, 5, 6, 8, 10, 11, 12, 13, 15
icad_postcode	Individual postcode, zip code		2, 3, 7, 8, 13, 21
icad_car	Number of cars family owns	0- none<br>1- One<br>2- Two +	3, 7, 13, 14, 16
Icad_house	Tenure of participants family home	1- owned<br>2- rented<br>3- other	1, 10, 12, 14, 16

* Studies that provided the variable. See key (Table S8) for each study code (1-21)

Table S6: Parental Information

ICAD Variable	Variable Descriptor	Coding	Studies*
icad_mother_height	Mothers height	CM	2, 3, 5, 6, 7, 8, 11, 13, 14, 15, 19
icad_mother_weight	Mothers weight	KG	2, 3, 5, 6, 7, 8, 11, 13, 14, 15, 19, 21
icad_father_height	Fathers height	CM	2, 3, 5, 6, 7, 8, 11, 13, 15, 19
icad_father_weight	Fathers weight	KG	2, 3, 5, 6, 7, 8, 11, 13, 15, 19, 21
icad_mother_ed	Mothers highest education	1- Up to & including high school<br>2- College vocational training<br>3- University +	1, 2, 3, 5, 6, 7, 8, 11, 13, 15, 17, 19, 20, 21
icad_father_ed	Fathers highest education	As above	1, 2, 3, 5, 6, 7, 8, 11, 13, 15, 17, 19, 20, 21
icad_mother_emp	Mothers employment status	1- Employed<br>2- Unemployed<br>3- Student<br>4- Carer , home duties<br>5- Retired<br>6- Absent due to illness<br>7- Other	1, 2, 3, 5, 7, 13, 21
icad_father_emp	Fathers employment status	As above	1, 2, 3, 5, 7, 13, 21

* Studies that provided the variable. See key (Table S8) for each study code (1-21)

Table S7: Other

ICAD Variable	Variable Descriptor	Coding	Studies*
icad_school	School codes	Three digit study code plus original school code provided by project	1, 2, 3, 4, 5, 6, 7, 9, 11, 13, 15, 16, 17, 18, 21
icad_sch_travel	Usual mode of transport TO school	1- car<br>2- bus<br>3- bike<br>4- walk	3, 5, 6, 11, 13, 14, 15, 16
icad_tv	Self-reported hours of TV watched per day	1- <1 hr<br>2- 1-2 hrs<br>3- 2-4 hrs<br>4- 4+ hours	3, 5, 6, 7, 8, 10, 11,12, 13, 14, 15
icad_comp	Self-reported hours of computer usage per day	1- <1 hr<br>2- 1-2 hrs<br>3- 2-4 hrs<br>4 - 4+ hours	3, 5, 6, 8, 10, 11, 12, 13, 14, 15
icad_house_comp	Number of children / siblings living with participant	0 – participant is only child in household<br>1 – one additional child in household<br>2-two additional children in household … (etc)	1, 2, 3, 7, 13, 14, 19, 21
icad_season	Season of measure 10 , 12 only	1- Nov 1st - April 30th<br>2- 1st May - 31st Oct	10, 12

* Studies that provided the variable. See key (Table S8) for each study code (1-21)

Table S8: ICAD Study Key

Study ID	Study Name	
1	Avon Longitudinal Study of Parents and Children	(ALSPAC)
2	Belgium Pre-School Study	
3	Children Living in Active Neigbourhoods	(CLAN)
4	Copenhagen School Child Intervention Study	(CSCIS)
5	Denmark European Youth Heart Study	(Denmark EYHS)
6	Estonia European Youth Heart Study	(Estonia EYHS)
7	Healthy Eating and Play Study	(HEAPS)
8	Iowa Bone Development Study	(IOWA)
9	Movement and Activity Glasgow Intervention in Children	(MAGIC)
10	National Health and Nutrition Examination Survey (2005.06)	(NHANES 2005-6)
11	Norway European Youth Heart Study	(Norway EYHS)
12	National Health and Nutrition Examination Survey (2005.06)	(NHANES 2003-4)
13	Personal and Environmental Associations with Children's Health	(PEACH)
14	Pelotas 1993 Birth Cohort	(Pelotas)
15	Portugal European Youth Heart Study	(Portugal EYHS)
16	Sport, Physical activity and Eating behavior: Environmental Determinants in Young people	(SPEEDY)
17	Project Trial of Activity for Adolescent Girls	(Project TAGG)
18	Children’s Health and Activity Monitoring for Schools (UK)	(CHAMPS UK)
19	Ballabeina Study	
20	Kinder-Sportstudie Study	(KISS)
21	CHAMPS: Physical Activity in Pre-school Children (US)	(CHAMPS US)
