# Supplementary material for: International children's accelerometry database (ICAD): Design and methods
Source: BMC Public Health. 2011 Jun 21;11:485. doi: 10.1186/1471-2458-11-485 (PMC3146860; doi:10.1186/1471-2458-11-485)
Supplement: Additional file 2 — Accelerometer Variables included in the International Children's Accelerometer Database (ICAD) [84-86] . [file 1471-2458-11-485-S2.PDF]

Additional File 2

Accelerometer Variables included in the International Children's Accelerometer Database (ICAD)

This document provides an overview of the accelerometer variables in the database. A more detailed data dictionary, providing a definition of all accelerometer variables in the ICAD database, can be found at http://www.mrc-epid.cam.ac.uk/Research/Studies/

Table S1: Introduction Variables (N=28)

Variable Name	Definition	Example
Reliability	Flags unreliable data. 0=Fine. 1= Spurious, 2=Temporally Shifted, 3= Questionable,	2
Device Name		Actigraph
Model	The generation (or model) of the accelerometer	GT1M
Version	The version of the model (not always provided)	2.2
Firmware	The firm wear used (not always provided)	v3.2.0
Serial Number	Where not available 9s have been inserted (e.g. 9999999999999)	lyn2b16061215
Epoch Period	i.e. 00:01:00 (i.e. 60 secs.)*	00:01:00
Header Start Date	The date that the device was initialized to start collecting data (as it appears in the header)	09/15/2006
Header Start Time	The time that the device was initialized to start collecting data (as it appears in the header)	05:00:00
Header Start Day of Week	The day of the week that the device was initialized to start collecting data (as it appears in the header)	Friday
Header Start Month	The month in which the device was initialized to start collecting data (as it appears in the header)	September
Header Download Date	The date that data was downloaded from the monitor (as it appears in the header)	09/22/2006

Header Age	This is obtained from a ‘read-in-file’ which specifies the truncated age (years) of the participant.	4
Read_InFileEnabled	Indicates whether a read-in-file (RIF)** was used	YES
Read_InFileStartDate	The start date on the RIF**.	04/12/2005
Read_InFileStartTime	The start time on the RIF**.	05:00:00
CollectionStartDay	The day that data is analyzed from.	1
CollectionStopDay	This is usually 7 days after ‘CollectionStartDay’	7
CollectionLength_indays	This is usually 7	7
DateFormatUsed	This indicates the data format used in the .DAT files	mm/dd/yyyy
RawDaysofCollection	These are the number of days of data collection	6.21
Position *(for Monday-Sunday)*	The order of data collection (e.g. Mon-Sunday allocated 1-7)	Day 5

* All data has been converted up to 60 seconds; ** An example of a Read-in-file (RIF) is found in Figure S1.

Figure S1: An example of a read-in-file (RIF). Read-in-files are used to a) Link age with the .DAT file (required for the age specific cutpoints) and b) to alter the start dates and/or times of individual .DAT files when necessary.

```
File   Edit   Format   View   Help
file name=AAb0000002.DAT, age=8, date format=mm-dd-yyyy, start date=09-18-1998, start time=05:00:00
file name=AAb0000002.DAT, age=8, date format=mm-dd-yyyy, start date=10-12-1998, start time=05:00:00
file name=AAb0000008.DAT, age=8, date format=mm-dd-yyyy, start date=09-18-1998, start time=05:00:00
file name=AAb0000012.DAT, age=8, date format=mm-dd-yyyy, start date=09-30-1998, start time=05:00:00
file name=AAb0000015.DAT, age=8, date format=mm-dd-yyyy, start date=09-30-1998, start time=05:00:00
file name=AAb0000018.DAT, age=8, date format=mm-dd-yyyy, start date=09-18-1998, start time=05:00:00
file name=AAb0000018.DAT, age=8, date format=mm-dd-yyyy, start date=09-18-1998, start time=05:00:00
file name=AAb0000020.DAT, age=8, date format=mm-dd-yyyy, start date=09-23-1998, start time=05:00:00
file name=AAb0000022.DAT, age=8, date format=mm-dd-yyyy, start date=09-18-1998, start time=05:00:00
file name=AAb0000028.DAT, age=8, date format=mm-dd-yyyy, start date=09-18-1998, start time=05:00:00
file name=AAb0000031.DAT, age=8, date format=mm-dd-yyyy, start date=09-18-1998, start time=05:00:00
file name=AAb0000038.DAT, age=7, date format=mm-dd-yyyy, start date=10-08-1999, start time=05:00:00
file name=AAb0000029.DAT, age=8, date format=mm-dd-yyyy, start date=09-18-1998, start time=05:00:00
file name=AAb0000051.DAT, age=8, date format=mm-dd-yyyy, start date=09-30-1998, start time=05:00:00
file name=AAb0000053.DAT, age=8, date format=mm-dd-yyyy, start date=09-18-1998, start time=05:00:00
file name=AAb0000081.DAT, age=8, date format=mm-dd-yyyy, start date=09-30-1998, start time=05:00:00
file name=AAb0000070.DAT, age=8, date format=mm-dd-yyyy, start date=10-12-1998, start time=05:00:00
file name=AAb0000080.DAT, age=8, date format=mm-dd-yyyy, start date=09-23-1998, start time=05:00:00
file name=AAb0000082.DAT, age=8, date format=mm-dd-yyyy, start date=09-30-1998, start time=05:00:00
file name=AAb0000082.DAT, age=7, date format=mm-dd-yyyy, start date=11-17-1999, start time=05:00:00
file name=AAb0000092.DAT, age=8, date format=mm-dd-yyyy, start date=09-18-1998, start time=05:00:00
file name=AAb0000095.DAT, age=8, date format=mm-dd-yyyy, start date=09-18-1998, start time=05:00:00
file name=AAb0000111.DAT, age=8, date format=mm-dd-yyyy, start date=09-23-1998, start time=05:00:00
file name=AAb0000112.DAT, age=8, date format=mm-dd-yyyy, start date=09-30-1998, start time=05:00:00
file name=AAb0000118.DAT, age=8, date format=mm-dd-yyyy, start date=09-23-1998, start time=05:00:00
file name=AAb0000121.DAT, age=8, date format=mm-dd-yyyy, start date=10-12-1998, start time=05:00:00
file name=AAb0000128.DAT, age=8, date format=mm-dd-yyyy, start date=10-07-1998, start time=05:00:00
file name=AAb0000139.DAT, age=8, date format=mm-dd-yyyy, start date=09-23-1998, start time=05:00:00
file name=AAb0000120.DAT, age=8, date format=mm-dd-yyyy, start date=10-12-1998, start time=05:00:00
file name=AAb0000122.DAT, age=8, date format=mm-dd-yyyy, start date=09-23-1998, start time=05:00:00
file name=AAb0000125.DAT, age=8, date format=mm-dd-yyyy, start date=10-07-1998, start time=05:00:00
file name=AAb0000129.DAT, age=8, date format=mm-dd-yyyy, start date=09-23-1998, start time=05:00:00
file name=AAb0000150.DAT, age=8, date format=mm-dd-yyyy, start date=10-07-1998, start time=05:00:00
file name=AAb0000151.DAT, age=8, date format=mm-dd-yyyy, start date=09-30-1998, start time=05:00:00
file name=AAb0000152.DAT, age=8, date format=mm-dd-yyyy, start date=09-30-1998, start time=05:00:00
file name=AAb0000182.DAT, age=8, date format=mm-dd-yyyy, start date=09-23-1998, start time=05:00:00
file name=AAb0000175.DAT, age=8, date format=mm-dd-yyyy, start date=09-23-1998, start time=05:00:00
file name=AAb0000177.DAT, age=8, date format=mm-dd-yyyy, start date=10-07-1998, start time=05:00:00
file name=AAb0000179.DAT, age=8, date format=mm-dd-yyyy, start date=10-12-1998, start time=05:00:00
```

Table S2: Wear Variables (N=378)

	By 7 days (i.e. Mon-Sun)	By 24 hour windows (i.e. 0-23hrs)for 7 days	2 time Windows (i.e. 15:30-16:00 and 15:30-16:30) for 7 days
Wear Counts	7	168	14
Wear Minutes	7	168	14
**TOTAL**	**14**	**336**	**28**

N.B. Non-wear is calculated as >60 minutes of consecutive zeros, allowing for 2 minutes of interruptions. This is provided for every day of monitoring (i.e. whether deemed valid or not)

Table S3: Intensity Variables (N=4347) (see Table 5 for Intensity Threshold Key)

	By 7 days (i.e. Mon-Sun)	By 24 hour windows (i.e. 1-24hrs) for 7 days	2 time Windows (i.e. 15:30-16:00 and 15:30-16:30) for 7 days
Motionless	7	168	14
Sedentary_Evenson	7	168	14
Sedentary_Pate	7	168	14
Sedentary_ Van Cauwenberghe	7	168	14
Light	7	168	14
Light_Evenson	7		14
Light_Pate	7	168	14
Light_ Van Cauwenberghe	7	168	14
Light_Trost(REG)	7	168	14
Light_Trost(REG)	7	168	14
Moderate	7	168	14
Moderate_Evenson	7	168	14
Moderate_Pate	7	168	14
Moderate_ Van Cauwenberghe	7	168	14
Moderate_Trost(REG)	7		14
Moderate_Trost(ELE)	7		14
Vigorous	7	168	14
Vigorous_Evenson	7	168	14
Vigorous_Pate	7	168	14
Vigorous_ Van Cauwenberghe	7	168	14
Vigorous_Trost(REG)	7	168	14
Vigorous_Trost(ELE)	7	168	14
MVPA	7	168	14
MVPA_Evenson	7	168	14
MVPA_Pate	7	168	14
MVPA_ Van Cauwenberghe	7	168	14
MVPA_Trost(REG)	7	168	14
MVPA_Trost(ELE)	7	168	14
MVPA_liberal	7	168	14
LVPA	7	168	14
**TOTAL**	**210**	**5040**	**420**

MVPA=Moderate-to vigorous physical activity, LVPA = Light-to-vigorous physical activity, METS= Metabolic Equivalent; REG = Regular; ELE = Elevated METS (see table S5).

Table S4: Accumulation Variables (N=3024) (see Table 5 for Intensity Threshold Key)

Accumulation	Accumulation Category	By 7 days (i.e. Mon-Sun)	By 24 hour windows (i.e. 0-23hrs) for 7 days	2 time Windows (i.e. 15:30-16:00 and 15:30-16:30) for 7 days
Sedentary_Evenson	1-30 min	7	168	14
Sedentary_Evenson	30-60 min	7	168	14
Sedentary_Evenson	60+ min	7	168	14
Sedentary_Pate	1-30 min	7	168	14
Sedentary_Pate	30-60 min	7	168	14
Sedentary_Pate	60+ min	7	168	14
Sedentary_ Van Cauwenberghe	1-30 min	7	168	14
Sedentary_ Van Cauwenberghe	30-60 min	7	168	14
Sedentary_ Van Cauwenberghe	60+ min	7	168	14
MVPA	1-10 min	7	168	14
MVPA	10+ min	7	168	14
MVPA_Evenson	1-10 min	7	168	14
MVPA_Evenson	10+ Min	7	168	14
MVPA_Pate	1-10 min	7	168	14
MVPA_Pate	10+ Min	7	168	14
MVPA_ Van Cauwenberghe	1-10 min	7	168	14
MVPA_ Van Cauwenberghe	10+ Min	7	168	14
MVPA_liberal	1-10 min	7	168	14
MVPA liberal	10+ min	7	168	14
MVPA_Trost (REG)	1-10 min	7	168	14
MVPA_Trost (REG)	10+ Min	7	168	14
MVPA_Trost (ELE)	1-10 min	7	168	14
MVPA_Trost (ELE)	10+ Min	7	168	14
**TOTAL**		**161**	**3864**	**322**

MVPA=Moderate-to vigorous physical activity; REG = Regular; ELE = Elevated METS (see table S5); METS= Metabolic Equivalent

Table S5: Intensity threshold Key

Cutpoint Title	Age Range	Start Value (>=)	Start Value Type	End Value (<)	End Value Type	Reference
Motionless	N/A	0.0	Counts	1.0	Counts	N/A
Sedentary	N/A	0.0	Counts	100.0	Counts	N/A
Light	N/A	100.0	Counts	3000.0	Counts	N/A
Moderate	N/A	3000.0	Counts	6000.0	Counts	N/A
Vigorous	N/A	6000.0	Counts		Counts	N/A
MVPA	N/A	3000.0	Counts			N/A
Light_Trost(REG)	6-18	100.0	Counts	3.0	METS	[84]
Moderate_Trost(REG)	6-18	3.0	METS	6.0	METS	[84]
Vigorous_trost(REG)	6-18	6.0	METS			[84]
MVPA_Trost (REG)	6-18	6.0	METS			[84]
Light_Trost(ELE)	6-18	100	Counts	4.0	METS	[84]
Moderate_Trost(ELE)	6-18	4.0	METS	7.0	METS	[84]
Vigorous_Trost(ELE)	6-18	7.0	METS			[84]
MVPA_Trost (ELE)	6-18	4.0	METS			[84]
MVPA_liberal	6-18	2000.0	Counts			N/A
Sedentary_ Van Cauwenberghe	5 and 6	0	Counts	1492	Counts	[85]
Light_ Van Cauwenberghe	5 and 6	1492	Counts	2340	Counts	[85]
Moderate_ Van Cauwenberghe	5 and 6	2340	Counts	3524	Counts	[85]
Vigorous_ Van Cauwenberghe	5 and 6	3524	Counts			[85]
MVPA_ Van Cauwenberghe	3-6	2340	Counts			[85]
Sedentary_Pate	5-8	0.0	Counts	152	Counts	
Light_Pate	5-8	152	Counts	1676	Counts	[43,63,86]
Moderate_Pate	5-8	1677	Counts	3364	Counts	[43,63,86]

Vigorous_Pate	5-8	3365	Counts			[43,63,86]
MVPA_Pate	5-8	>1677	Counts			[43,63,86]
Light_Evenson	5-8	100	Counts	2295	Counts	[47]
Moderate_Evenson	5-8	2296	Counts	4011	Counts	[47]
Vigorous_Evenson	5-8	4012	Counts			[47]
MVPA_Evenson	5-8	2296	Counts			[47]

MVPA=Moderate-to vigorous physical activity; METS= Metabolic Equivalent; REG = Regular; ELE = Elevated METS
